# Novel Mutations in ACVR1 Result in Atypical Features in Two Fibrodysplasia Ossificans Progressiva Patients

**DOI:** 10.1371/journal.pone.0005005

**Published:** 2009-03-30

**Authors:** Kirsten A. Petrie, Wen Hwa Lee, Alex N. Bullock, Jenny J. Pointon, Roger Smith, R. Graham G. Russell, Matthew A. Brown, B. Paul Wordsworth, James T. Triffitt

**Affiliations:** 1 Institute of Musculoskeletal Sciences, Botnar Research Centre, Nuffield Department of Orthopaedic Surgery, University of Oxford, Oxford, United Kingdom; 2 Structural Genomics Consortium, Nuffield Department of Clinical Medicine, ORCRB, University of Oxford, Oxford, United Kingdom; 3 The University of Queensland, Diamantina Institute for Cancer Immunology and Metabolic Medicine, Princess Alexandra Hospital, Woolloongabba, Queensland, Australia; University of Cambridge, United Kingdom

## Abstract

Fibrodysplasia Ossificans Progressiva (FOP) is a rare, heritable condition typified by progression of extensive ossification within skeletal muscle, ligament and tendon together with defects in skeletal development. The condition is easily diagnosed by the presence of shortened great toes and there is severe advancement of disability with age. FOP has been shown to result from a point mutation (c.617G>A) in the *ACVR1* gene in almost all patients reported. Very recently two other mutations have been described in three FOP patients. We present here evidence for two further unique mutations (c.605G>T and c.983G>A) in this gene in two FOP patients with some atypical digit abnormalities and other clinical features. The observation of disparate missense mutations mapped to the GS and kinase domains of the protein supports the disease model of mild kinase activation and provides a potential rationale for phenotypic variation.

## Introduction

Fibrodysplasia Ossificans Progressiva (FOP) is a rare, autosomal dominant disease with complete penetrance involving the progressive ossification of the skeletal muscles, fasciae, tendons and ligaments. Smooth muscle and cardiac muscles remain unaffected. Due to low reproductive fitness the condition is mainly a result of spontaneous new mutations and it has a prevalence of approximately one in two million individuals worldwide. FOP shows no geographic, ethnic, racial or gender predisposition [1].

Individuals with FOP appear normal at birth except for great toe abnormalities; these being short, deviated and later monophalangic. Extensive fusion of the lateral masses of the cervical vertebrae is often seen with hypoplasia of the vertebral bodies. Femoral necks are abnormally wide and there may be true bone exostoses additional to muscle ossification with short malformed thumbs being less common [2], [3]. Following periodic acute episodes of mysositis, endochondral ossification of striated muscles generally begins in the occipital, cervical and upper paraspinal muscles and later affects most muscles around the major joints. Minor trauma or viral illnesses can initiate acute inflammatory mysositic episodes leading to progressive heterotopic ossification, which is amplified by surgical intervention or removal. The phenotype of FOP is affected by both genetic and environmental factors with postnatal heterotopic ossification varying with life history and environmental exposure [4]. Ossification occurs progressively over the course of a lifetime in an inevitable episodic and unpredictable manner with most patients being confined to a wheelchair by the third decade of life and requiring lifelong care.

Recently the genetic cause of FOP was discovered within the *ACVR1* gene, which encodes a type I bone morphogenetic protein (BMP) transmembrane receptor [5]. A single point mutation (c.617G>A) was identified in all FOP patients studied. This non-synonymous mutation causes an Arg206His amino acid substitution within the GS (glycine-serine rich) domain of the ACVR1 protein, and has been confirmed by our further work and also by others [6], [7]. Type I receptors such as ACVR1 are normally inactive until binding of extracellular BMP stimulates their phosphorylation by type II receptors within the GS domain. This activates the type I protein to recruit and phosphorylate Smad signalling molecules within the cell that subsequently cause gene transcription or repression. The GS domain is also negatively regulated through the binding of FKBP12, which provides a mechanism to buffer the overall signal in the cell [8]. The switch between the inactive and active states has been illustrated by crystal structures of the type I TGF-beta receptor kinase domain as well as its complex with FKBP12 [9], [10].

It has been observed that the Arg206His mutation causes the ACVR1 protein to be mildly constitutively active [11]. Since the mutation occurs in the activating GS domain it has been suggested that this promotes a shift in the absence of BMP towards the active kinase structure causing over-activation of ACVR1 and subsequent R-Smad signalling cascades [5], [12]. Recently other ACVR1 mutations have been reported in three FOP patients. A Japanese man with FOP has been reported with an *ACVR1* mutation, c.1067G>A resulting in a Gly356Asp amino-acid change in the protein kinase domain [13]. In two Italian patients, a novel mutation c.774G>C, leading to the Arg258Ser substitution in the kinase domain of the ACVR1 receptor was seen. In a three-dimensional model of the protein structure, Arg258 maps in close proximity to the GS domain [14]. Here we report two patients with expression of the major characteristics that define FOP, but with some atypical features, that both lack the typical, specific c.617G>A mutation and the other recently reported mutations but present two different and separate coding mutations in *ACVR1*.

## Methods

### Patients

Patient 1 is female and was diagnosed clinically with FOP in 2003 aged 14 years. The first presentation was with a painful bony lump over her right scapula after a fall and clinical examination showed that there was only one short great toe with the other normal. Subsequently she developed multiple tender bony swellings, and the detection of toe abnormality confirmed the diagnosis. Her right shoulder was fixed in internal rotation. Fixed flexion deformities of both elbows were present. Her lumbar spinal movements were restricted. The patient continued to have frequent flares of the condition with increased inflammatory lesions over her shoulder joints, neck, and jaw and fusion of the neck within 6 months of clinical presentation. This progressive formation of lesions which later ossify is characteristic to FOP. However, the relatively late age of onset is unusual and the malformation of only one great toe has yet to be documented in another patient.

Patient 2 is a 52 year old female with FOP whose clinical features were first reported in 1976 [15] and subsequently reviewed later [15], [16]. Severe reduction deformities in all digits were noted at birth. Her first presentation of the disease was with lumps, usually painful, on the occiput. By 6 years the patient had a stiff spine and shoulders. By 14 years both elbows and the right hip showed ectopic ossification. At 18 years the left hip showed ectopic ossification and at 20 years there was ossification around the jaw after dental extraction. At 26 years the patient had complete spinal fixation, the shoulders were fixed in adduction, the elbows fixed in flexion, hip movement restricted and fixed in slight flexion and the jaw gape was 0.3cm. She showed mild cognitive impairment. In addition, there was diffuse scalp hair thinning beginning at 14 years of age.

### Genetic Analyses

Patient samples. Blood samples were collected from patients and family members and from normal controls with informed consent. Lymphoblastoid cell lines were a generous gift from Professor J M Connor, Glasgow,UK.

DNA Isolation. Genomic DNA was isolated from EBV-transformed cell lines or from peripheral blood samples using the FlexigeneTM Kit (Qiagen Ltd, UK) according to the manufacturer's instructions. The resulting DNA was resuspended in the elution buffer supplied in the kit.

Identification of mutations. PCR primers (MWG Biotech AG, Edersberg, Germany) were designed to amplify exon 6 of *ACVR1* which contains the c.617G>A mutation. The sample DNA was amplified using PCR with optimized MgCl2 concentrations and annealing temperatures using reagents from Sigma, UK. Amplified DNA was purified on filter plates (Millipore (UK) Ltd, Watford, UK) and sequenced in both forward and reverse directions with Big Dye version 3 using an automated sequencer (ABI3100, Applied Biosystems, Warrington, UK). Subsequently primers were designed for all 11 exons of *ACVR1* which were sequenced in both patients. To ensure that the mutations found in the patients were those specifically present in patients with FOP 100 healthy controls were screened also. The mutation in patient 2 created a restriction site for the restriction enzyme StyI and controls were screened for this site using a restriction fragment length polymorphism. In patient 1, the mutation did not create a restriction site and all individuals were screened by sequencing exon 6 of *ACVR1* as described above.

Restriction fragment length polymorphism. The restriction enzyme StyI (New England Biolabs, Hitchin, UK) was used to digest amplified *ACVR1* exon 8 in patient 2 and healthy controls. The reaction was left overnight at 37°C and analyzed by agarose gel electrophoresis with ethidium bromide staining and a 100bp ladder (New England Biolabs, Hitchin, UK).

### Homology modelling

The crystal structure of the kinase domain of the type I TGF-beta receptor (PDB code 1B6C) in complex with FKBP12 [9] was used as a template to model ACVR1. The alignment was performed with the program ICM [17] and used for homology modelling of the native ACVR1 sequence using the same software. The resulting model was then energy minimised and side-chain movements were allowed to resolve clashes. Using this native model, in silico mutations were introduced corresponding to those described in this study. The mutation Gly328Glu required limited loop sampling to resolve main-chain clashes resulting from the insertion of a larger side chain. Models were visually inspected before selection. Side-chain rotamer optimisation was performed for all mutations as well as for neighbouring residues. Electrostatic potential isosurfaces as implemented in ICM were calculated for all the resulting models.

## Results and Discussion

**Figure 1:**
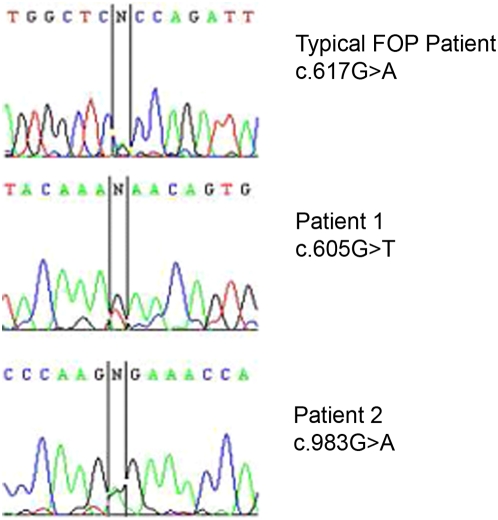
DNA sequencing electropherograms of a typical FOP patient, and of atypical patients 1 and 2, at the positions of the causative ACVR1 mutations.

**Figure 2:**
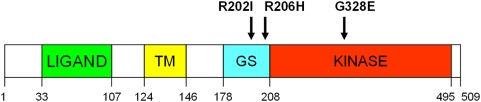
A schematic of ACVR1 domain organization showing the position of mutations in patients 1 (Arg202Ile) and 2 (Gly328Glu) with respect to the classical mutation (Arg206His).

The previously described c.617G>A mutation has been confirmed in most UK FOP patients with characteristic features of FOP, but was not present in either of the two patients described here (Figure 1). Patient 1 is heterozygous for the novel mutation c.605G>T in *ACVR1*. Like the c.617G>A mutation, this mutation is found within the GS domain of the ACVR1 protein, but results in a new substitution Arg202Ile. Patient 2 is heterozygous for the novel mutation c.983G>A found within *ACVR1*, which results in a Gly328Glu substitution. This mutation occurs outside the GS region in the kinase catalytic domain (Figure 2).

To date there are no available crystal structures for the kinase domain of ACVR1. To understand the structural implications of the new mutations, we built homology models of the native and mutated ACVR1 kinase domains using the closest available structure of the TGF-beta receptor (PDB code 1B6C). Importantly, this TGF-beta receptor domain shares the same mechanism of regulation and is highly similar in sequence to ACVR1 (66% sequence identity) with no insertion or deletions in the aligned sequence. Structural models must be interpreted carefully as disease mutations can alter the wild-type structure.

**Figure 3:**
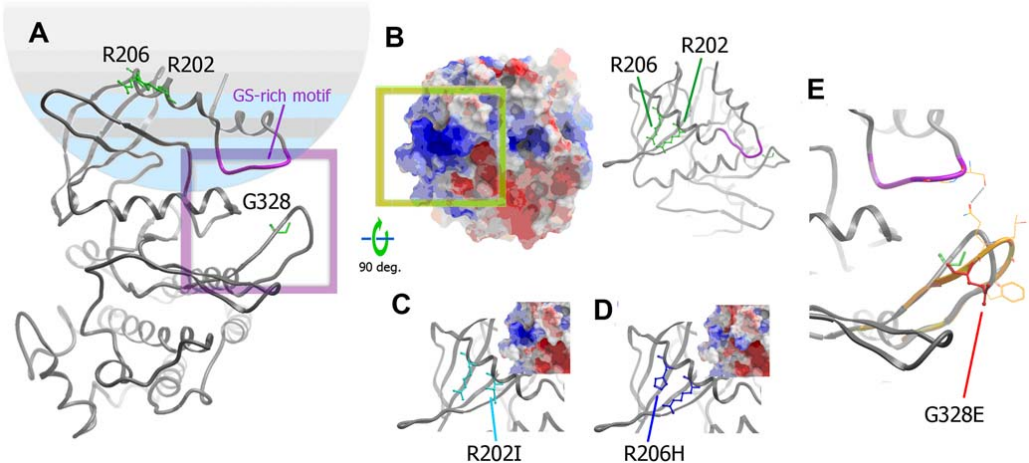
Homology models of ACVR1. (A) Wild-type ACVR1 kinase domain. The residues where mutations are described in this study are represented as sticks (green, labelled). A ribbons representation of the GS-rich motif is highlighted in magenta. A purple frame marks the zoomed area in panel E. (B) wild-type ACVR1 model rotated 90° around the X-axis to show the surface occluded upon binding of FKBP12 (shown both as ribbons and surface coloured according to electrostatic potential). The green box denotes the positive patch seen in the model of wild-type ACVR1. (C) and (D) mutations Arg202Ile and Arg206His are shown as ribbons, with the mutations indicated (same view as panel B). The predicted electrostatic potential for each mutant protein is shown in the insert (framing is equivalent to the green box of panel B). (E) Mutation Gly328Glu induces a significant conformational change in the loop where it is sited. One of the putative conformations is depicted in orange (wild-type loop conformation shown in grey). In this example a potential direct interaction could be formed between the modelled loop and the GS-rich motif.

The mutated residues in patient 1 and classical FOP patients map to the same region in the ACVR1 model positioned in the αGS2 helix immediately following the glycine-serine (GS) rich loop. (Fig. 3A–D). The native residues, both arginines, are solvent accessible, and their mutation will directly impact upon the electrostatic property of the GS domain surface. In particular, the Arg202Ile mutation occurs within the recognition site of ACVR1 for its inhibitor FKBP12. The αGS2 helix also packs above the kinase L45 loop which determines Smad interaction. The striking similarity to the classical FOP mutation is consistent with the current disease model suggesting that FOP results from increased kinase activity [11]. It is noteworthy that patient 1 has less severe clinical features than a typical FOP patient. Interestingly, the electrostatic potential is changed less by the substitution Arg202Ile (patient 1) than by Arg206His (classical), when both are compared to wild-type ACVR1 (Fig. 3B–D). Furthermore, Arg206 shows greater interaction with the L45 loop including an invariant salt bridge with Asp269. These subtle effects may be correlated with the phenotypic differences.

The mutation c.983G>A (amino acid change Gly328Glu) presented by patient 2 maps to the loop following helix αE in the kinase domain, but is positioned in the three-dimensional structure adjacent to the GS-rich loop (Fig. 3A, E). In this substitution the introduction of an acidic residue (glutamic acid) will again change the local electrostatic potential. The native loop sequence is absolutely conserved between ACVR1 and the TGF-beta receptor ensuring a reliable initial model. Here the structure adopts a well formed hairpin-like loop and the introduction of a bulkier side chain will force a local change in conformation (Fig. 3E). The precise conformation (or dynamics) of the new loop is not trivial to predict preventing reliable interpretation of a change in functional state. Potentially, any perturbation could weaken GS-domain interactions that maintain the inactive kinase conformation.

In conclusion, two unique mutations in the *ACVR1* gene have been detected in two FOP patients from the UK with some atypical digit abnormalities and other clinical features. The resultant mutations are likely to result in local structural changes in the ACVR1 protein as revealed by interrogating homology models of the native and mutated ACVR1 kinase domains. In particular, the electrostatic surface potential of the ACVR1 GS domain is predicted to be appreciably affected by these disparate point mutations, promoting a shift in the equilibrium between the inactive and active ACVR1 structures causing mild kinase activation. The ACVR1 receptors would thus show reduced ligand-dependence and result in variable receptor activity effects causing the different phenotypic features observed. The identified mutants present new targets for ACVR1 kinase inhibitors that have shown potential to manage heterotopic ossification [18], [19].
